# Mcm10: A Dynamic Scaffold at Eukaryotic Replication Forks

**DOI:** 10.3390/genes8020073

**Published:** 2017-02-17

**Authors:** Ryan M. Baxley, Anja-Katrin Bielinsky

**Affiliations:** Department of Biochemistry, Molecular Biology, and Biophysics, University of Minnesota, Minneapolis, MN 55455, USA; baxle002@umn.edu

**Keywords:** CMG helicase, DNA replication, genome stability, Mcm10, origin activation, replication initiation, replication elongation

## Abstract

To complete the duplication of large genomes efficiently, mechanisms have evolved that coordinate DNA unwinding with DNA synthesis and provide quality control measures prior to cell division. Minichromosome maintenance protein 10 (Mcm10) is a conserved component of the eukaryotic replisome that contributes to this process in multiple ways. Mcm10 promotes the initiation of DNA replication through direct interactions with the cell division cycle 45 (Cdc45)-minichromosome maintenance complex proteins 2-7 (Mcm2-7)-go-ichi-ni-san GINS complex proteins, as well as single- and double-stranded DNA. After origin firing, Mcm10 controls replication fork stability to support elongation, primarily facilitating Okazaki fragment synthesis through recruitment of DNA polymerase-α and proliferating cell nuclear antigen. Based on its multivalent properties, Mcm10 serves as an essential scaffold to promote DNA replication and guard against replication stress. Under pathological conditions, Mcm10 is often dysregulated. Genetic amplification and/or overexpression of *MCM10* are common in cancer, and can serve as a strong prognostic marker of poor survival. These findings are compatible with a heightened requirement for Mcm10 in transformed cells to overcome limitations for DNA replication dictated by altered cell cycle control. In this review, we highlight advances in our understanding of when, where and how Mcm10 functions within the replisome to protect against barriers that cause incomplete replication.

## 1. Efficient Replication of Large Eukaryotic Genomes

At a speed of 1.5 kb per minute, it would take approximately 60 days to duplicate one copy of the human genome if a single, unidirectional fork replicated each chromosome. To rapidly generate a complete copy of the genome, replication is initiated from numerous origins distributed across each chromosome where the number of initiation sites appears to be related to genome size [1,2,3,4,5,6,7]. In budding yeast, ~400 replication origins are activated to copy a genome of ~1.2 × 10^7^ bp, whereas the significantly larger human genome contains ~5 × 10^4^ origins to duplicate a genome of 3 × 10^9^ bp [1,2,3,4,5,6,7]. Importantly, the number of origins licensed for replication initiation exceeds the number utilized during a normal S-phase [8,9,10]. These unfired or “dormant” origins serve as backup sites for initiation in the event of replication stress to ensure that DNA replication can be completed [11,12]. Interestingly, the average distance between replication origins is only moderately increased in humans in comparison to yeast, as both are in the range of 30–60 kb [6,13,14,15,16]. However, the maximum region replicated by a single origin, or replicon, in humans (up to ~5 Mb) is orders of magnitude larger than in yeast (up to 60 kb) [6,13,14,15,16]. Therefore, different challenges exist in lower and higher eukaryotes to warrant replication fidelity and maintain genome integrity.

In all eukaryotes, replication begins with the loading of the catalytic core of the replicative helicase, which is composed of the minichromosome maintenance complex proteins 2-7 (Mcm2-7). Unlike in eukaryotic viruses, helicase loading and activation are temporally separated into two distinct stages. The first step, origin licensing, occurs via loading of Mcm2-7 double hexamers onto double-stranded DNA (dsDNA) [17,18,19,20]. This is achieved during late mitosis and G1-phase through the coordinated action of the origin recognition complex (ORC), cell division cycle 6 protein (Cdc6), and Cdc10-dependent transcript 1 (Cdt1) to complete assembly of the pre-replication complex (pre-RC) [19,20,21,22]. Once a sufficiently high number of replication origins have been licensed [23], cells prohibit formation of additional pre-RCs and commit to the second stage of DNA replication, origin firing and DNA synthesis [18,24,25,26]. To this end, the helicase co-factors cell division cycle 45 (Cdc45) and go-ichi-ni-san (GINS) are recruited to chromatin [18,24,25,26,27,28]. Finally, to initiate DNA synthesis, Cdc45-Mcm2-7-GINS (CMG) helicase dimers are activated and physically separate to proceed in a bidirectional manner [18,24,25,26]. Minichromosome maintenance protein 10 (Mcm10) participates in this activation process and remains physically attached to the Mcm2-7 complex throughout DNA replication [29,30,31,32,33,34,35,36,37]. In this review, we focus on Mcm10 and how it ensures timely and accurate completion of DNA replication.

## 2. Discovery and Biochemical Characterization of Mcm10

Mcm10 is an evolutionarily conserved component of the eukaryotic replication machinery [38,39]. The *MCM10* gene was identified in two independent genetic screens in *Saccharomyces cerevisiae*. Initially uncovered over 30 years ago as a temperature sensitive allele of *DNA43* defective in both entry and completion of S-phase [40,41], a second screen revealed additional *mcm10/dna43* mutants that were unable to maintain minichromosomes [42,43]. Investigations in many eukaryotic model organisms including fission yeast (*Schizosaccharomyces pombe*), nematodes (*Caenorhabditis elegans*), fruit flies (*Drosophila melanogaster*), frogs (*Xenopus laevis*), zebrafish (*Danio rerio*), mice (*Mus musculus*), and humans (*Homo sapiens*) have revealed *MCM10* homologs [31,44,45,46,47]. Much of the core replication machinery, including Mcm10, is also conserved in plants [48]. Curiously, *Drosophila* but not human Mcm10 was able to functionally complement a *mcm10* mutant in budding yeast [35,45,46]. These observations imply that despite its conserved structure and role in DNA replication, it is important to determine organism specific details of Mcm10 function. Finally, Mcm10 homologs have not been found in bacteria or archaea, showing that *MCM10* is unique within eukaryotic genomes [38,39,49,50,51].

Despite the lack of catalytic domains indicative of enzymatic function, Mcm10 associates with replication origins, facilitates their activation and becomes part of the replisome [30,35,37,52,53,54]. Several studies have identified structural motifs in Mcm10 that associate with linear single-stranded (ss-) and dsDNA, as well as more complex topological structures [33,51,55,56,57]. Furthermore, distinct regions direct interactions between Mcm10 and several replication factors, including the Mcm2-7 complex [32,34,43,45,58,59,60], Cdc45 [45,55,61], DNA polymerase alpha (Pol-α) [30,57,62,63,64,65], ORC [45,46,58,66], proliferating cell nuclear antigen (PCNA) [67], Chromosome transmission fidelity 4 (Ctf4) [65,68] and RecQ like helicase 4 (RecQL4) [69]. These data support a model in which Mcm10 coordinates helicase activity with DNA synthesis through interactions with different protein complexes at the replication fork [39,50,51]. Below, we review the current understanding of Mcm10’s functional domains that facilitate these interactions.

**Figure 1:**
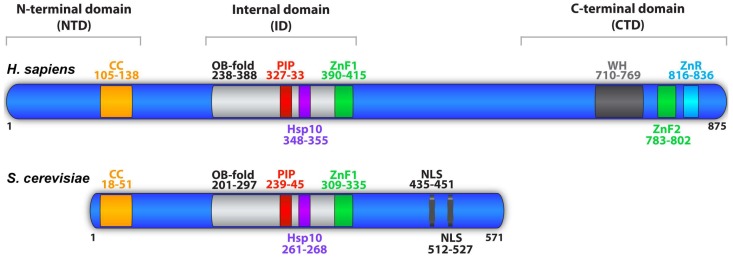
The domain structure of minichromosome maintenance protein 10 (Mcm10). Full-length Mcm10 is depicted for *Homo sapiens* (875 amino acids (aa)) and *Saccharomyces cerevisiae* (571 aa). Mcm10 functional domains and the amino acid regions they span depicted. The N-terminal domain (NTD) contains a coiled-coil (CC, orange) motif responsible for Mcm10 self-interaction. The internal domain (ID) mediates Mcm10 interactions with proliferating cell nuclear antigen (PCNA) and DNA polymerase-alpha (Pol-α) through a PCNA-interacting peptide (PIP) box (red) and Hsp10-like domain (purple), respectively. These motifs reside in the oligonucleotide/oligosaccharide binding (OB)-fold (light gray). The OB-fold along with zinc-finger motif 1 (ZnF1, green) serve as a DNA-binding domain. The C-terminal domain (CTD) is specific to metazoa and interacts with DNA primarily through ZnF2 (green). The CTD also includes the zinc ribbon (ZnR, blue) and winged helix motif (WH, dark gray); however their functions are currently unknown. A bipartite nuclear localization sequence (NLS) has been identified in *S. cerevisiae*.

**Figure 2:**
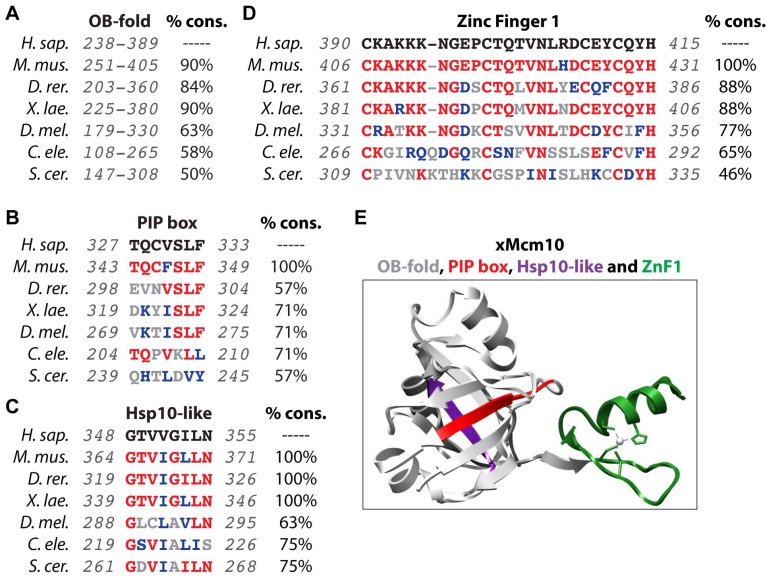
Evolutionary conservation of functional domains in the Mcm10 ID. (**A**–**D**) Comparison of the amino acid sequences from *Homo sapiens*, *Mus musculus*, *Danio rerio*, *Xenopus laevis*, *Drosophila melanogaster*, *Caenorhabditis elegans*, *Saccharomyces pombe* and *Saccharomyces cerevisiae* of the OB-fold (**A**), PIP box (**B**), Hsp10-like (**C**) and Zinc-Finger 1 (**D**) domains. The full sequence alignment for the OB-fold is not shown due to size constraints, but can be found in Warren et al., [70]. The percent conservation (% cons.), defined as the percentage of amino acid positions identical (in red) or strongly similar (in blue) to those of human Mcm10, is listed for each domain sequence. The total region aligned for each sequence listed in gray. (**E**) The crystal structure of the *Xenopus* Mcm10 (xMcm10) OB-fold (gray), PIP box (red), Hsp10-like (purple) and Zinc-Finger 1 (green) domains is shown. The structure was generated using pdb data file 3EBE and the Chimera program (http://www.cgl.ucsf.edu/chimera) [83].

Biochemical analyses and sequence alignment of Mcm10 homologs have revealed three major structural regions. Referred to as the N-terminal (NTD), internal (ID) and C-terminal domains (CTD), each contains distinct functional regions involved in DNA binding and/or protein-protein contacts (Figure 1) [38,39,51]. The ID is the most highly conserved region of Mcm10 and mediates both protein-DNA and protein-protein interactions (Figure 1 and Figure 2). DNA binding occurs via two motifs: a canonical oligonucleotide/oligosaccharide-binding fold (OB-fold) and a single CCCH zinc-finger (ZnF1) (Figure 1 and Figure 2) [57,62,63,70]. Unlike other proteins carrying these motifs, the Mcm10 OB-fold and ZnF1 are in a unique configuration and form a continuous interaction surface [57], capable of binding ss- and dsDNA [33,51,57,70,71,72]. Mcm10 does not have a preference for particular DNA sequences or topological structures, but its affinity for ssDNA is higher than for dsDNA [33,51,55,56,57]. In addition to DNA binding motifs, the ID contains specific sites that contact Pol-α, PCNA and Mcm2-7 (Figure 1) [30,43,45,46,51,57,58,59,60,63,67,70]. Association with Pol-α occurs via a hydrophobic patch termed the heat shock protein 10 (Hsp10)-like domain [30,57,63,70], whereas PCNA binds to a noncanonical PCNA interacting peptide (PIP) box, QxxM/I/LxxF/YF/Y (Figure 2) [39,67]. Notably, the putative PCNA interaction motif in higher eukaryotes bears close resemblance to the QLsLF consensus binding site for the prokaryotic β-clamp, which functions similarly to PCNA in promoting polymerase processivity [39,50,73]. Both the Hsp10-like domain and PIP box lie within the OB-fold on perpendicular β-strands (Figure 1), suggesting that Pol-α and PCNA compete with each other. However, Pol-α can be easily displaced by ssDNA [57].

**Figure 3:**
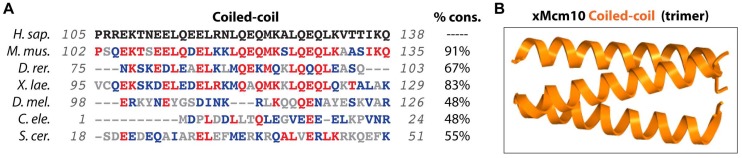
Evolutionary conservation of functional domains in the Mcm10 NTD. (**A**) Comparison of the amino acid sequences from *H. sapiens*, *M. musculus*, *D. rerio*, *X. laevis*, *D. melanogaster*, *C. elegans*, *S. pombe* and *S. cerevisiae* of the coiled-coil domain. The percent conservation (% cons.), defined as the percentage of amino acid positions identical (in red) or strongly similar (in blue) to those of human Mcm10, is listed for each domain sequence. The total region aligned for each sequence listed in gray. (**B**) The crystal structure of the *Xenopus* Mcm10 (xMcm10) coiled-coil domain is shown. The structure was generated using pdb data file 4JBZ and the Chimera program (http://www.cgl.ucsf.edu/chimera) [83].

The NTD is common among Mcm10 proteins from yeast to humans, but is not essential and less well conserved than the central ID (Figure 1 and Figure 3) [74,75]. Functionally, the NTD contributes to self-oligomerization and partner protein interaction [39,50]. Homocomplex formation of *Xenopus* and human Mcm10 clearly depends on the NTD [55,72,75]. A conserved coiled-coil (CC) domain within the NTD mediates dimer and trimer formation of purified *Xenopus* Mcm10 (Figure 1 and Figure 3) [51,75]. Human Mcm10 was proposed to form trimers or a hexameric ring, with the latter reinforced by electron microscopy reconstructions and model fitting based on the archaeal Mcm helicase and simian virus 40 large T-antigen [55,72]. However, the electron density map of the high-resolution crystal structure of *Xenopus* Mcm10 is not fully compatible with ring formation, leaving the true nature of the Mcm10 homo-oligomer open for further exploration [38,55,70,72]. Furthermore, current data lack insight regarding how a hexameric Mcm10 ring would be loaded onto DNA. These discrepancies notwithstanding, oligomerization of Mcm10 agrees with the characterization of *S. cerevisiae* Mcm10 complexes that associate with DNA [30,56]. The stoichiometry of DNA binding by Mcm10 is 1:1 on dsDNA, but 3:1 on ssDNA [56], suggesting that oligomerization may be triggered by DNA unwinding. Mcm10 oligomerization would thus present an elegant solution to the problem that ssDNA evicts Pol-α from the OB-fold [57]. Finally, the NTD promotes resistance to replication stress, as failure to oligomerize dramatically increases sensitivity to hydroxyurea in checkpoint deficient cells [74]. Independent of its role in oligomerization, the first 150 amino acids of the NTD interact with mitosis entry checkpoint 3 (Mec3), a component of the yeast radiation sensitive 9 (Rad9), hydroxyurea sensitive 1 (Hus1), radiation sensitive 1 (Rad1) checkpoint clamp referred to as 9-1-1 [74]. It appears that Mcm10 promotes resistance to UV irradiation in budding yeast through direct binding of the 9-1-1 clamp, whereby it might stabilize stalled replication forks [74].

**Figure 4:**
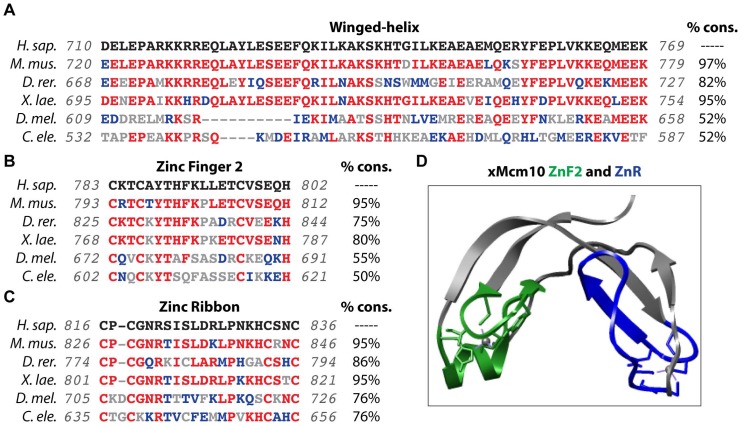
Evolutionary conservation of functional domains in the Mcm10 CTD. (**A**–**C**) Comparison of the amino acid sequences from *H. sapiens*, *M. musculus*, *D. rerio*, *X. laevis*, *D. melanogaster* and *C. elegans* of the Winged Helix (**A**), Zinc-Finger 2 (**B**) and Zinc-Ribbon (**C**). The percent conservation (% cons.), defined as the percentage of amino acid positions identical (in red) or strongly similar (in blue) to those of human Mcm10, is listed for each domain sequence. The total region aligned for each sequence listed in gray. (**D**) The crystal structure of the *Xenopus* Mcm10 (xMcm10) Zinc-Finger 2 (green) and Zinc-Ribbon (blue) domains is shown. The structure was generated using pdb data file 2KWQ and the Chimera program (http://www.cgl.ucsf.edu/chimera) [83].

The Mcm10 CTD, although not present in unicellular eukaryotes, is conserved among metazoan species from nematodes to humans (Figure 1 and Figure 4). The CTD contains a winged helix domain (WH) and two zinc chelating motifs, a CCCH zinc-finger (ZnF2) and a CCCC zinc-ribbon (ZnR) (Figure 1 and Figure 4). ZnF2 is required for the CTD to bind DNA, but the function of the ZnR has not been clearly defined, although it shares homology with the ZnRs found in archaeal and vertebrate Mcm proteins [39,51,55,57,76]. Mutation of the ZnR disrupts archaeal double hexamer formation, whereas alteration of the ZnR in budding yeast Mcms reduces viability [76,77,78,79], suggesting that it may mediate protein-protein interactions important for proper helicase function. Recent analysis of *Drosophila* Mcm10 demonstrated that the CTD directs interaction with heterochromatin protein 1a (HP1a) in vitro, a finding that is further supported by in situ proximity ligation [80]. This interaction is deemed important for cell cycle regulation and cell differentiation [80]. Furthermore, the CTD of human Mcm10 is necessary for nuclear localization although a bona fide NLS has not been defined [81]. Interestingly, the budding yeast C-terminus carries two bipartite nuclear localization signals (NLSs) that are each sufficient for directing Mcm10 to the nucleus (Figure 1), however, a homologous region is not present in metazoan Mcm10 [82]. Recent work from two independent groups has also mapped the major Mcm2-7 interaction surface, via Mcm2 and Mcm6, to a portion of Mcm10’s C-terminus in budding yeast. Again, this particular region is not conserved in higher eukaryotes [32,34]. Functionally, the CTD is similar to the ID, specifically in mediating interactions with DNA and Pol-α [51,55,62]. The DNA binding surfaces in the ID and CTD can be utilized simultaneously, as *Xenopus* Mcm10 binds in vitro with approximately 100-fold higher affinity than either domain individually [51]. Finally, DNA binding of the ID and CTD can be modulated by acetylation and this will be further discussed below [62].

## 3. The Multifaceted Regulation of Mcm10 Function

Mcm10 is regulated via changes in expression, localization and post-translational modification. The E2F/Rb (retinoblastoma) pathway, which is central to normal cell cycle control and proliferation, regulates transcription of *MCM10* in human HCT116 cells [84,85]. Furthermore, an essential E3 ubiquitin ligase, retinoblastoma binding protein 6 (RBBP6), ubiquitinates and destabilizes the transcriptional repressor zinc finger and BTB domain-containing protein 38 (ZBTB38) thereby relieving inhibition of *MCM10* transcription [86,87]. Interestingly, RBBP6 (also known as PACT or P2P-R) interacts with the critical cell cycle regulators Rb and p53 to modulate cell cycle progression [86,88,89]. Furthermore, the zinc-finger transcription factor GATA-binding factor 6 (GATA6) promotes *MCM10* expression in highly proliferative mouse follicle progenitor cells by stimulating Ectodysplasin-A receptor-associated adapter protein (Edaradd) and NF-κB signaling [90]. *MCM10* expression levels are also controlled by microRNAs, such as miR-215, which directly regulates *MCM* as well as other cell cycle genes, including *MCM3* and *CDC25A* [91,92]. This suggests coordinated suppression of genes that promote proliferation. Finally, *MCM10* expression is often increased in rapidly proliferating tumor cells (discussed in more detail below), pointing to a potential role in not just facilitating but actively driving cell cycle progression.

In addition to controlling *MCM10* expression, several post-translational modifications regulate Mcm10 turnover or modulate the activity of functional domains. Cellular levels of human Mcm10 increase as the cell cycle approaches the G1/S boundary and decrease in late G2/M-phase [93,94,95]. In HeLa and U2OS cell lines, Mcm10 depletion during mitosis is proteasome dependent [93,95]. The oscillation of Mcm10 levels is similar to other cell cycle regulators whose degradation is mediated by the ubiquitin-proteasome pathway [96]. Mcm10 is a substrate of the cullin 4 (Cul4), damaged DNA binding 1 (DDB1), viral protein R binding protein (VprBP) E3 ubiquitin ligase (Table 1) [81,95,97,98]. These observations are consistent with the role of the cullin-RING E3 ligase family in regulating multiple cell cycle and DNA replication related proteins [99]. Although Mcm10 contains substrate recognition motifs for the anaphase promoting complex/cyclosome (APC/C), it is not an APC/C target [95]. The described degradation mechanism is also activated in response to high doses of UV-radiation, likely to stall DNA replication instantaneously [81]. Furthermore, in response to human immunodeficiency virus 1 (HIV-1) infection, viral protein R (VPR) enhances the proteasomal degradation of endogenous Cul4-DDB1-VprBP substrates, including Mcm10, which causes G2/M arrest [98]. Lastly, ubiquitination of Mcm10 has also been observed in budding yeast, although this modification does not appear to drive protein degradation, but rather regulates Mcm10 function during S-phase (Table 1) [67,100].

Besides ubiquitination, phosphorylation of Mcm10 is also important for its functional regulation. In HeLa cells, the phosphorylation of Mcm10 is proposed to facilitate release from chromatin [93]. Subsequently, several high-throughput proteomics studies have identified a large number of putative phosphorylation sites on Mcm10 [101,102,103,104,105,106,107,108,109,110,111,112]. To date there has not been additional validation or functional characterization of these phosphorylation sites, although 23 have been reported in multiple datasets (Table 1) [101]. Interestingly, *Xenopus* Mcm10 is phosphorylated on various S-phase cyclin-dependent kinase (S-CDK) target sites [113]. Of the seven sites identified (Table 1), only serine 630 is conserved in other metazoa [113]. Recombinant *Xenopus* S630A mutant protein that cannot be phosphorylated supports chromatin loading and bulk DNA synthesis but significantly reduces replisome stability in vitro [113]. Decreased fork stability also leads to increased DNA damage following treatment with the topoisomerase inhibitor camptothecin [113]. The homologous site in human Mcm10 (S644) has been reported in the human phosphoproteome database, and warrants further investigation [101,102,106]. Future studies will be important to clarify our understanding of how phosphorylation may regulate Mcm10 in different biological systems.

In addition to Mcm10 regulation by phosphorylation and ubiquitination, acetylation modulates the DNA binding properties of human Mcm10. In vitro assays and in vivo analyses (in HCT116 cells) provide evidence that the ID and CTD of Mcm10 can be acetylated by the p300 acetyltransferase at more than 20 lysines (Table 1) [62]. Sirtuin 1 (SIRT1), a member of the sirtuin family of deacetylases and homolog of yeast Sir2, can deacetylate a subset of these residues [62]. Intriguingly, acetylation increases the DNA binding affinity of the ID but decreases affinity of the CTD in vitro [62]. Furthermore, the depletion of SIRT1 leads to increased levels of total and chromatin-bound Mcm10, disruption of the replication program, DNA damage and G2/M arrest [62]. Taken together, these observations suggest that acetylation of Mcm10 might regulate protein levels and dynamically controls the overlapping functions of the ID and CTD in DNA association or protein binding.

## 4. Mcm10 is a Central Player in Multiple Steps of DNA Replication

Mcm10 is an essential regulator of DNA replication initiation. Early evidence for this came from 2D gel analyses in yeast that reported decreased firing of two specific origins (ORI1 and ORI121) in temperature-sensitive *mcm10-1* mutants [43]. In *S. cerevisiae*, Mcm10 is loaded onto chromatin in G1 and remains bound during S-phase [30]. One clear pre-requisite for Mcm10 chromatin binding is pre-RC assembly, as association of Mcm10 with origins of replication is dependent on the Mcm2-7 complex [29,30,31,32,33,34]. Studies utilizing a Mcm10-degron system found that depletion during G1-phase prevented a significant number of cells from initiating DNA synthesis [30,114,115]. Building on these reports, the timing and mechanism of Mcm10’s role in replication initiation remains a topic of active research. 

At licensed origins, DNA replication is initiated through a multi-step process. Helicase activation requires that the Dbf4-dependent kinase Cdc7 (DDK) and S-CDK phosphorylate several targets [116,117,118,119]. DDK-dependent phosphorylation of Mcm2-7 initiates recruitment of synthetically lethal with *dpb11 3* (Sld3), its binding partner Sld7, and the helicase co-activator Cdc45 [116,117,120,121]. Similarly, S-CDK-dependent phosphorylation of Sld2 and Sld3 initiates recruitment of helicase co-activator GINS and the pre-loading complex (pre-LC), consisting of Sld2, DNA polymerase B II 11 (Dpb11) and DNA polymerase epsilon (Pol-ε) [116,117,119,120,121]. Next, the origin is unwound to allow recruitment of Pol-α/primase to ssDNA [52,122,123] and as the CMG helicase progresses, it generates larger ssDNA regions that are protected by the replication protein A (RPA) complex [24,124]. DNA synthesis begins with the production of RNA-DNA primers by Pol-α/primase on both strands [122,123] and requires frequent re-priming for Okazaki fragment synthesis [18,125,126]. During replication elongation, these primers are extended on the leading strand by Pol-ε and on the lagging strand by DNA polymerase delta (Pol-δ) [24,122,123], in association with PCNA, the trimeric replication clamp [24,127]. The process of replication requires Mcm10 at several steps, and three major functions have been proposed. First, Mcm10 is necessary for recruitment of GINS and Cdc45 to complete assembly of the CMG helicase. Second, following CMG assembly Mcm10 is needed for activation of the helicase. Third, after origin unwinding Mcm10 is required for polymerase loading to initiate DNA synthesis. The following paragraphs will evaluate these roles in more detail.

## 5. Mcm10 Promotes Assembly of the Replicative Helicase

Investigations of Mcm10’s role in CMG complex assembly have largely focused on stable association of Cdc45. Early studies in *Xenopus* egg extracts reported that Cdc45 binding was significantly reduced following depletion of Mcm10 [31]. A similar observation was made in fission yeast, as Mcm10 degradation in vivo resulted in the loss of nuclear Cdc45 following detergent wash [61,128]. In agreement, stable association of the CMG complex was reduced and chromatin loading of Cdc45 and Sld5 were not detected following small interfering RNA (siRNA) knockdown of Mcm10, RecQL4 or Ctf4 in HeLa cells [129]. These data imply that Mcm10 might be integral for CMG assembly. However, there is evidence that loss of Mcm10 does not abolish Cdc45 recruitment, as CMG formation in S-phase eventually recovers to wild type levels [33,61,128]. Taken together, these studies support the hypothesis that Mcm10 deficiency delays recruitment and/or decreases stability of Cdc45 interaction with the replicative helicase. However, there are also several reports consistent with a model in which Mcm10 is dispensable for CMG assembly. Two independent groups utilizing inducible Mcm10 degradation in budding yeast found no effect on chromatin association of Cdc45 [30,115]. These data are in agreement with the finding that depletion of Mcm10 from purified S-phase extracts does not reduce Cdc45 recruitment [130]. This also holds true in a reconstituted system with 16 purified yeast replication factors [131]. 

Delineating the timing of Mcm10 loading with respect to DDK and S-CDK activities has provided additional insights regarding Mcm10’s placement in CMG assembly. After formation of the pre-RC, origin activation requires DDK phosphorylation of Mcm2-7, followed by S-CDK phosphorylation of Sld2 and Sld3 [130,132,133]. Experiments using whole cell extracts from yeast reported that the action of DDK followed by S-CDK was essential for Mcm10 recruitment, as Mcm10 was undetectable when S-CDK treatment was performed first [130]. However, in a minimal in vitro system with purified proteins, CMG formation and DNA synthesis occurred regardless of which kinase was added to the reaction first [131]. It seems possible that S-CDK targets may become rapidly dephosphorylated by phosphatases present in the yeast extracts used by Heller and colleagues [130], and that therefore S-CDK activity is required immediately before Mcm10 recruitment. In fact, there is supporting evidence for this notion [131,134]. Overall, these studies agree that robust Mcm10 recruitment occurs following kinase activated CMG assembly. However, they are not in agreement with experiments in fission yeast that reported Mcm10-dependent stimulation of DDK activity, thereby placing Mcm10 at the replisome early in CMG assembly [60]. These latter findings are consistent with recent results in budding yeast in which Cdc45 recruitment to DNA is facilitated by DDK-dependent (via phospho-Sld3) and DDK-independent (via Mcm10) mechanisms [33]. A possible solution to this apparent discrepancy is presented below.

**Figure 5:**
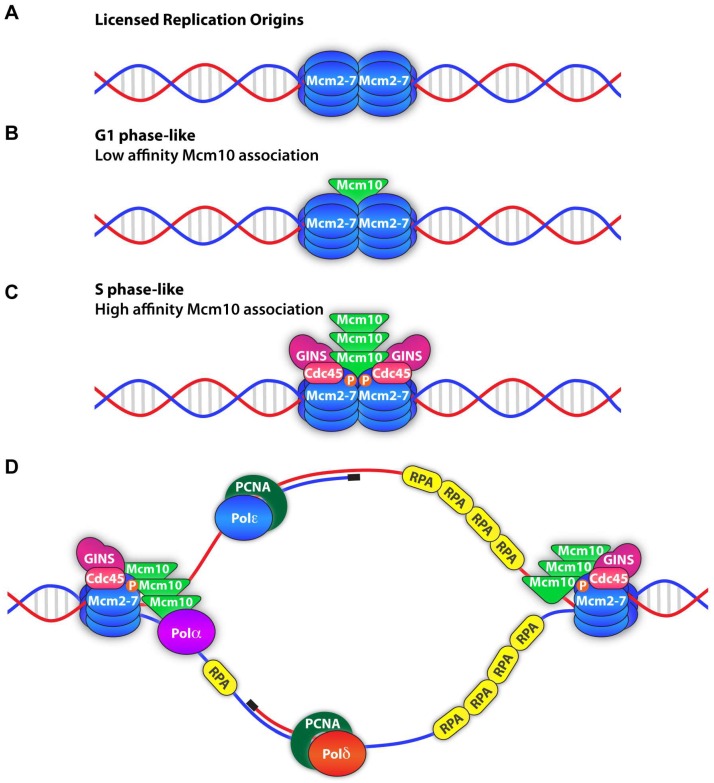
Model of the association of Mcm10 with the replisome in initiation and elongation. (**A**) A Mcm2-7 double hexamer is loaded onto dsDNA and represent a licensed replication origin. (**B**) Mcm10 directly interacts with the Mcm2-7 with low affinity in G1-phase-like binding prior to CMG assembly. (**C**) High affinity binding of Mcm10 to the Mcm2-7 complex in S-phase like binding takes place with formation of the CMG complex. (**D**) Following helicase activation, replication forks progress in opposite directions from each origin. Mcm10 binds and stabilizes ssDNA (right fork) and is later replaced by RPA. Mcm10 loading of DNA polymerase-alpha (Pol-α) (left fork) is repeatedly needed to generate RNA/DNA primers (black DNA regions) for Okazaki fragment synthesis. Processive DNA polymerization is executed by DNA polymerase-epsilon (Pol-ε) (extending the blue leading strand) and DNA polymerase-delta (Pol-δ) (extending the red lagging strand).

Studies by the Diffley and Lou laboratories investigating Mcm10 recruitment to the CMG complex may provide the best compromise to reconcile the conflicting data discussed above [32,34]. Both reports highlight the requirement for the C-terminal ~100 amino acids of yeast Mcm10 to directly bind to Mcm2-7 double hexamers [32,34]. This interaction permits both a low affinity “G1-phase-like” and high affinity “S-phase-like” binding of Mcm10 to Mcm2-7. The “G1-phase-like” binding seems consistent with mass spectrometry analysis of replication reactions that detect Mcm10 on DNA independently of DDK activity, but at levels 10–100 fold lower than other firing factors [134]. Therefore, Mcm10 may initially associate with the pre-RC prior to Cdc45 addition, and then bind more robustly at later stages of CMG assembly (Figure 5) [32,34].

## 6. Activation of the CMG Helicase Relies on Mcm10

Replication initiation begins with origin unwinding to generate ssDNA that is encircled by one CMG helicase complex, which then translocates in 3′ to 5′ direction [18,24,39,135]. Early studies found that depletion of Mcm10 from *Xenopus* extracts resulted in the inability to unwind a double stranded plasmid and recruit RPA to chromatin [31]. A similar deficiency in RPA recruitment was demonstrated following depletion of Mcm10 in budding and fission yeast [33,114,115,136]. As RPA is the major ssDNA-binding complex in eukaryotes, this provides strong evidence that dsDNA unwinding is impaired in the absence of Mcm10. This is generally in agreement with the notion that Mcm10 is one of the key origin “firing factors” identified via mass spectrometry in yeast replication complexes [134]. Importantly, in a reconstituted budding yeast replication system, Mcm10 both promotes RPA loading and is essential for DNA synthesis [131]. Two independent but not mutually exclusive mechanisms exist for Mcm10 in CMG activation. First, Mcm10 may actively promote remodeling of the replicative helicase from a double to a single CMG complex. Observations that Mcm10 stimulates DDK activity prior to CMG assembly (discussed above) and recruits replisome components required for initiation, such as the human Sld2 homolog RecQL4 support this model [69,129,137,138,139]. Second, Mcm10 may stabilize ssDNA following DNA unwinding prior to RPA association. This idea is strengthened by numerous experimental observations. Mcm10 preferentially binds to ssDNA rather than dsDNA [51,55,56,57,71], and the disruption of ZnF1 in fission yeast impaired RPA recruitment to replication origins [136]. Furthermore, analysis of a *S. cerevisiae mcm10* mutant defective in DNA binding showed significantly decreased RPA association at specific origin sequences, and a severe decline in viability [71]. Moreover, viability of this *mcm10* mutant could not be enhanced by a *mcm5* mutation (*mcm5^bob-1^*) that bypasses the requirement for DDK-dependent phosphorylation of Mcm2 [140,141,142]. These observations strongly support a critical role for Mcm10 in stabilizing the replisome during origin firing through binding of newly exposed ssDNA, rather than a stimulatory function in DDK-dependent Mcm2 phosphorylation. In this model, Mcm10 holds on to ssDNA first, but is later evicted by RPA, which protects longer regions of ssDNA behind the progressing helicase. This is also consistent with the fact that that RPA has an apparent 40-fold higher affinity for ssDNA than Mcm10 [143]. This mechanism would then allow Mcm10 to remain anchored to the Mcm2-7 complex and travel with the replisome [30,35,37,52,53].

## 7. Mcm10-Dependent Polymerase Loading

Unperturbed DNA synthesis in eukaryotes relies on three DNA polymerases. The recruitment of Pol-ε occurs prior to DNA unwinding, via interactions with the GINS complex, and is independent of Mcm10 [130,144,145]. However, Mcm10 is an important player in polymerase loading during replication elongation. Experiments in budding and fission yeast, *Xenopus* egg extracts and human cells all demonstrated that Mcm10 facilitates chromatin loading of Pol-α to initiate Okazaki fragment synthesis [18,30,64,65,130,146]. Mcm10 likely works in concert with the cohesion factor Ctf4, which forms a homo-trimeric hub [29,65], fitting with the fact that Mcm10 forms a homo-trimeric scaffold [51,55,75]. It should be noted, however, that budding yeast Ctf4 is dispensable for DNA replication in vivo and in vitro [131,147], strongly arguing that in *S. cerevisiae* Mcm10 is the critical connector between DNA polymerization and helicase activities [30]. Furthermore, *Xenopus* Mcm10 interacts with acidic nucleoplasmic DNA-binding protein 1 (And-1)/Ctf4 to initiate DNA replication [65]. In human cells, RecQL4 promotes interactions between Mcm10 and And-1/Ctf4 consequently facilitating efficient DNA replication [129,137,138]. 

Following Pol-α loading, Mcm10 directly interacts with the replication clamp PCNA. Disruption of this interaction via a single amino acid substitution within Mcm10’s PIP-motif causes lethality in *S. cerevisiae* [67]. This protein-protein interaction is dependent on diubiquitination of Mcm10, which is proposed to make the internally located PIP motif accessible for PCNA binding [67]. Interestingly, diubiquitination occurs during G1/S-phase and disrupts Mcm10’s interaction with Pol-α [67]. Therefore, ubiquitination of Mcm10 following primer synthesis by Pol-α could function to recruit PCNA and facilitate loading onto primed DNA [39,50,67]. Interestingly, recruitment of the lagging strand polymerase Pol-δ was reduced following Mcm10 depletion in budding yeast [130]. One explanation of these data is that without Mcm10-dependent generation of ssDNA regions and recruitment of Pol-α to initiate DNA synthesis, PCNA loading is decreased. Impaired PCNA recruitment could diminish Pol-δ association at the replication fork. Whether the Mcm10-PCNA interaction occurs in higher eukaryotes is currently unknown, although such an observation would strongly support a conserved role of Mcm10 in elongation. Of note, it was recently proposed that the PIP boxes identified in several PCNA interacting proteins may belong to a broader class of “PIP-like” motifs that have the ability to bind multiple target proteins [148]. In line with this idea, the yeast Mcm10 PIP motif is also important for direct binding to the Mec3 subunit of the 9-1-1 checkpoint clamp [74]. Thus, Mcm10’s direct interaction network that stabilizes the fork during normal DNA synthesis and in response to replication stress could extend beyond factors currently identified.

## 8. Replication Fork Progression and Stability Relies on Mcm10

Loss of Mcm10 causes replication stress and increased dependence on pathways that maintain genome integrity [149,150,151,152,153]. Genetic analyses in yeast have demonstrated that *mcm10* mutants rely on the checkpoint signaling factors mitosis entry checkpoint 1 (Mec1) and radiation sensitive 53 (Rad53) that are activated in response to RPA coated ssDNA [39,50,66,149,150]. Under conditions of high replication stress, Rad53 hyperactivation blocks S-phase progression [154,155]. However, moderate chronic replication stress in *mcm10-1* mutants under semi-permissive conditions only elicits low-level Rad53 activity and allows the cell cycle to advance. Under these circumstances, underreplicated DNA eventually triggers the mitotic spindle assembly checkpoint (SAC) [156,157]. To evade SAC activation when replication stress is tolerable, these cells rely on the E3 small ubiquitin-like modifier (SUMO) ligase methyl methanesulfonate sensitivity 21 (Mms21) and the SUMO-targeted ubiquitin ligase complex synthetic lethal of unknown (X) function 5/8 (Slx5/8) in order to progress through M-phase [157]. Overall, these studies suggest that moderate Mcm10 deficiency in budding yeast primarily causes defects in replication fork progression. Indeed, experiments using *mcm10-1* mutants found that the DNA synthesis and growth defects at non-permissive temperatures could be alleviated by mutations in *mcm2* [39,43,50,59,63,67,150]. In addition, loss-of-function mutations in *mcm5* and *mcm7* also suppressed *mcm10-1* mutant phenotypes [59]. The simplest interpretation of these data is that *mcm* mutations disrupt helicase activity, slow fork progression and reduce ssDNA accumulation, thus suppressing checkpoint activation in *mcm10* mutants.

In metazoa, Mcm10 is also important for replication fork progression and stability. Two independent siRNA screens identified Mcm10 as a potent suppressor of chromosome breaks and incomplete replication [6,152,153]. Knockdown experiments in HeLa cells revealed defects in DNA synthesis that resulted in late S-phase arrest, suggesting that cells accumulate significant damage if replication proceeds with reduced Mcm10 levels [158,159,160]. Recently, investigators have employed the DNA fiber technique to assess replication dynamics and measure inter-origin distance (IOD) as well as fork velocity. Interestingly, Mcm10 depletion decreased fork velocity in U2OS, but not in HCT116 cells, during unperturbed cell cycle conditions [62,87]. One explanation is that the intrinsically faster rate of synthesis in U2OS cells causes an increased requirement for Mcm10 to sustain fork speed. Surprisingly, both studies found that the IOD was decreased following siRNA knockdown of *MCM10*, indicative of an actual increase in origin firing [62,87]. Moreover, a recent study using *Xenopus* egg extracts also argued that Mcm10 depletion primarily affected elongation and not replication initiation [113,161]. In these studies, RPA loading occurred in the absence of more than 99% of Mcm10 and the efficiency of bulk DNA synthesis only decreased by 20% [113]. Consistent with a role in elongation, Mcm10 depletion in this system impaired replisome stability, as levels of PCNA, RPA, and several CMG components showed drastically reduced chromatin association [113,161]. Loss of replisome stability caused a markedly increased sensitivity to camptothecin and resulted in fork collapse and DSBs [113]. Several possibilities exist to reconcile these data with those that argue for an essential role in replication initiation. For example, origin firing may require very small amounts of Mcm10. In this scenario, even when Mcm10 is undetectable by western blot enough may remain on chromatin to facilitate initiation. Alternatively, dormant or backup origins, the majority of which are not activated during a normal cell cycle, could bypass the requirement for Mcm10. The ability of these origins to be activated via an alternative mechanism would support a role solely in replication elongation for Mcm10. It is our opinion that this is unlikely, based on the in vitro reconstruction of origin firing with purified proteins [131], but the issue is certainly a top priority to be resolved.

## 9. Emerging Connections between Mcm10 and Cancer Development

Several studies have found *MCM10* expression to be significantly upregulated in cancer cells [92,162,163,164,165,166]. A comparison of *MCM10* mRNA levels in normal and tumor samples on the Broad Institute Firebrowse gene expression viewer consistently shows higher abundance in cancer samples (www.firebrowse.org). Oncogene driven overexpression of *MCM10* was reported in a collection of neuroblastoma tumors and cell lines, as well as in Ewing’s sarcoma tumor cells [162,163]. Interestingly, *MCM10* overexpression increases with advancing tumor stage in cervical cancer [165] and correlates with the transition from confined to metastasized renal clear cell carcinoma [92]. Additional cell cycle related transcripts, including other MCM genes, are also upregulated in these cancer samples [92,162,163,164,165,166], suggesting that enhanced Mcm10 production may simply coincide with increased DNA synthesis. Contrary to this idea, *MCM10* has been proposed to be part of a group of high-priority genes that promote cell cycle related processes in cancer cells [167]. Moreover, a recent analysis of urothelial carcinomas found that the level of *MCM10* expression, but not of other *MCM* genes, was a highly significant predictor of both disease-free and metastasis-free survival [166]. In fact, increases in *MCM10* expression could be detected prior to histological changes [166]. Since high gene expression and protein production strongly correlates with negative outcomes, the detection of Mcm10 protein levels could be a valuable early indicator of progression in urothelial carcinomas [166]. Future investigations should determine whether early detection of increased Mcm10 production has prognostic value in other cancer types.

**Figure 6:**
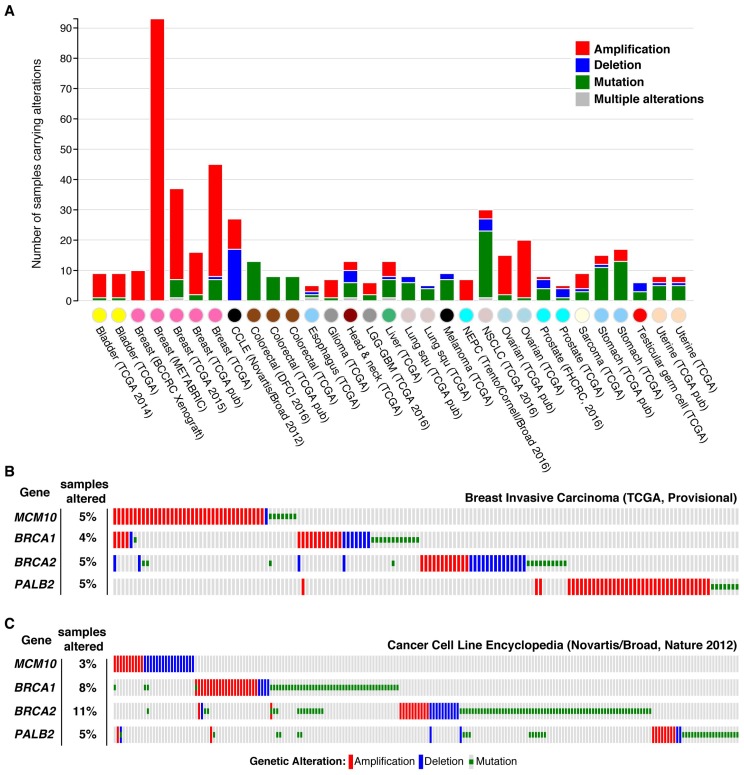
*MCM10* alterations in human cancer samples and exclusivity with BRCA-associated mutations. (**A**) Bar graph showing the number and class of alterations including amplifications (red), deletions (blue), mutations (green) or a combination (gray) of *MCM10* identified in different cancer types by multiple groups. The tissue/cell type and dataset for each column are listed on the x-axis. Only datasets with 5 or more *MCM10* alterations are shown. (**B**,**C**) Plots showing the overlap of genetic alterations including amplifications (red), deletions (blue) and mutations (green) in *MCM10* or breast cancer (BRCA) associated genes (*BRCA1, BRCA2, partner and localizer of BRCA2 (PALB2)*) in the Breast Invasive Carcinoma dataset (The Cancer Genome Atlas [TCGA]) (**B**) or the Cancer Cell Line Encyclopedia (Novartis/Broad) [171]. The data and depictions shown in this figure were accessed via and/or modified from information listed on the cBioPortal for Cancer Genomics (http://www.cbioportal.org/) [168,169].

In addition to transcriptional changes, analyses of cancer genomes have identified chromosomal amplifications, deletions and mutations in *MCM10* [39,50,168,169,170]. Current data indicate that over half (~54%) of the genetic alterations are amplifications, whereas ~35% are mutations and only ~11% are deletions [168,169]. The majority of mutations identified to date are missense mutations (93%), with the remainder roughly split between splicing (3.7%) and nonsense mutations (3.2%) [168,169]. Notably, a higher number of *MCM10* alterations have been identified in breast cancer samples than in other tumor types (Figure 6) [168,169]. These alterations are generally mutually exclusive with changes in the breast cancer (BRCA) susceptibility genes *BRCA1*, *BRCA2* or *partner and localizer of BRCA2* (*PALB2)* (Figure 6) [168,169]. This trend was maintained in a similar analysis of the Cancer Cell Line Encyclopedia dataset (Figure 6) [168,169,171]. These data suggest that alterations in two or more of these genes are not well tolerated. Experiments evaluating this hypothesis could prove valuable in the treatment of BRCA associated tumors. Taken together, these data clearly show that *mcm10* is altered in cancer genomes. What remains to be determined is whether these changes are causative or a consequence of oncogenesis, or whether mutations may simply be a byproduct of decreased genome stability seen in cancer cells.

Given the elevated Mcm10 levels [92,162,163,165,166] and frequency of genomic amplifications observed in cancer cells [168,169], it seems reasonable to propose that during oncogenesis cells rely on increased Mcm10 levels to ameliorate replication stress and drive cell cycle progression. Future evaluations of this hypothesis will be crucial to understanding Mcm10’s contribution to cancer development. However, this idea does not address the impact of gene deletions or loss-of-function mutations, such as truncations or amino acids substitutions that might disrupt important functional domains. Based on experimental observations, it seems possible that these genetic alterations could increase replication stress and DNA damage. Thus, these lesions likely occur late in oncogenesis after cells have already deactivated pathways that induce cell cycle arrest or apoptosis in response to sources of genome instability. Extending data from yeast, it will be interesting to understand whether there is an increased requirement for Ring finger protein 4 (RNF4), the human homolog of yeast Slx5/Slx8, [157,172], in order to promote survival under moderate levels of replication stress.

## 10. Conclusions

In the several decades since Mcm10 was first discovered, significant progress has been made in understanding its role in eukaryotic DNA replication. Nevertheless, active research across many laboratories continues to provide mechanistic insights into how Mcm10 stimulates replication initiation and promotes fork progression during elongation. These important cellular functions, when compromised, contribute to human disease. Based on recent studies, future investigations into Mcm10’s relationship with cancer development and progression could lead to discoveries with significant prognostic and even therapeutic value.

## Figures and Tables

**Table 1:** Post-translational modifications of Mcm10.

Modification	Role	Species/System	Region/Residue(s)	Enzyme	Reference(s)
Ubiquitination	Target for proteasome dependent degradation	Human Mcm10 (HeLa, U2OS) in vivo	440–525 783–803 843–875 (regions that can mediate degradation)	Cul4-DDB1-VprBP	[93,95,97,98]
Ubiquitination	Functional regulation during S-phase	Yeast Mcm10 (*Saccharomyces cerevisiae*)	K85, K122, K319, K372, K414, K436	Not identified	[67,100]
Phosphorylation	Unknown function	Human Mcm10 (HeLa)	T85, S93, S150, S155, A182, S203, S204, A210, S212, T217, R286, T296, S488, S548, S555, S559, S577, S593, Y641, S644, T663, S706, S824 (* only sites identified in more than 2 datasets are listed)	Not identified, except T85 which is ATR or ATM dependent.	[93,101,102,103,104,105,106,107,108,109,110,111,112]
Phosphorylation	Replisome stability	*Xenopus* extract	S154, S173, S206, S596, S630, S690, S693	S-CDK	[113]
Acetylation	Protein stability and DNA binding	Human Mcm10	K267, K312 *, K318, K390 *, K657, K664, K668, K674 *, K681 *, K682 *, K683 *, K685 *, K737 *, K739 *, K745 *, K761 *, K768 *, K783, K847 *, K849 *, K853, K868, K874	p300 (acetylase) SIRT1 * deacetylase) * indicates subset of SIRT1 target residues	[62]

Listed are the modifications identified for Mcm10 in different model systems, their functional role, protein region or specific residues modified, and the enzyme responsible, if determined. Abbreviations in this table include: minichromosome maintenance protein 10 (Mcm10), cullin 4-damaged DNA binding 1-viral protein R binding protein (Cul4-DDB1-VprBP), ataxia telangiectasia and Rad3-related protein (ATR), ataxia-telangiectasia mutated (ATM), S-phase cyclin dependent kinase (S-CDK), Sirtuin 1 (SIRT1).
